# Representation and Utilization of Laboratory Data in CT-Based Acute Abdominal Emergency Radiology: A Methodological Content Analysis

**DOI:** 10.3390/tomography12020024

**Published:** 2026-02-13

**Authors:** Betül Tiryaki Baştuğ, Türkan Güney

**Affiliations:** 1 Department of Radiology, Faculty of Medicine, Bilecik Şeyh Edebali University, Bilecik 11230, Turkey; 2 Department of Biochemistry, Faculty of Medicine, Bilecik Şeyh Edebali University, Bilecik 11230, Turkey; turkan.guney@bilecik.edu.tr

**Keywords:** acute abdominal emergencies, computed tomography, laboratory data, diagnostic integration, emergency radiology, radiology reporting

## Abstract

Background: Acute abdominal emergencies represent a major diagnostic challenge in emergency medicine, requiring rapid and accurate integration of clinical, laboratory, and imaging data. Although laboratory parameters play a central role in real-world diagnostic workflows, the extent to which they are systematically represented and integrated within radiology research publications remains unclear. Objective: To evaluate how laboratory data are represented, contextualized, and functionally utilized in radiology publications focusing on computed tomography (CT)–based evaluation of acute abdominal emergencies. Methods: A methodological content analysis was conducted on 72 original radiology research articles published between 2020 and 2024. Eligible studies focused on CT-based imaging of acute abdominal emergency conditions. Publications were screened and analyzed at the title and abstract level using a predefined coding framework to assess the presence of laboratory data, types of laboratory parameters reported, their contextual role (background information, imaging trigger, diagnostic modifier, or prognostic indicator), degree of laboratory–imaging integration, and presence of decision-oriented reporting. Descriptive statistics were used to summarize reporting patterns. Results: Laboratory data were reported in 61.1% of all included studies (*n* = 44/72). However, their functional utilization varied substantially. Laboratory parameters were most frequently presented as background clinical information, whereas explicit use as imaging triggers (26.4%, *n* = 19/72), diagnostic modifiers (19.4%, *n* = 14/72), or components of explicit laboratory–imaging integration (15.3%, *n* = 11/72) was less common. Decision-oriented reporting was present in 23.6% of all studies (*n* = 17/72). Explicit integration was described in publications addressing diagnostically complex and time-sensitive conditions, such as acute bowel ischemia and severe acute pancreatitis. Conclusion: Laboratory data are commonly reported in CT-based radiology publications addressing acute abdominal emergencies; however, the manner in which these data are incorporated into imaging-centered diagnostic narratives varies across studies. Differences are observed in how laboratory–imaging relationships are described, with some publications presenting integrated discussion and others reporting imaging findings independently of laboratory context. These observations characterize reporting practices within the analyzed literature and do not imply statistical associations or causal effects.

## Simple Summary

Acute abdominal emergencies are common and time-critical conditions in emergency medicine, where laboratory tests and computed tomography (CT) are routinely used together to support diagnosis and management. In clinical practice, laboratory abnormalities often guide imaging decisions and influence diagnostic confidence. However, it remains unclear whether this integrated diagnostic approach is consistently reflected in radiology research publications. In this study, we analyzed recent CT-based radiology articles on acute abdominal emergencies to evaluate how laboratory data are reported and used alongside imaging findings. We found that laboratory results are frequently mentioned but are often limited to background information and are less commonly integrated into imaging interpretation or decision-making. Improving laboratory–imaging integration in radiology publications may enhance clinical relevance and interdisciplinary communication.

## 1. Introduction

Acute abdominal emergencies represent one of the most challenging domains in emergency radiology, requiring rapid and accurate diagnostic decision-making under time-sensitive conditions. Clinical entities such as acute appendicitis, bowel obstruction, perforated viscus, acute pancreatitis, diverticulitis, acute cholecystitis, and mesenteric ischemia frequently overlap in symptomatology, limiting the reliability of isolated clinical assessment [1,2]. In this context, cross-sectional imaging—particularly computed tomography (CT)—has become the cornerstone of diagnostic evaluation due to its high sensitivity, broad anatomical coverage, and ability to detect life-threatening complications [3,4,5].

Beyond imaging, laboratory parameters constitute an important complementary component of the diagnostic workflow. Biomarkers such as leukocyte count, C-reactive protein (CRP), serum lactate, amylase, lipase, and liver enzymes may provide early physiological indicators of inflammation, ischemia, infection, or organ dysfunction, and can contribute to imaging referral, urgency, and interpretation [6,7,8]. For example, elevated serum lactate may increase clinical suspicion of mesenteric ischemia [9,10] and prompt urgent CT angiography, whereas marked hyperamylasemia or lipase elevation directs diagnostic attention toward pancreatic pathology and provides context for CT findings in acute pancreatitis [11].

Despite this recognized interplay, the integration of laboratory data within radiological reporting and imaging-focused publications remains variable. The radiology literature has traditionally emphasized morphological imaging findings, while laboratory results are frequently presented as background clinical information rather than as explicitly integrated diagnostic elements [12,13]. Although structured reporting initiatives have improved standardization of imaging descriptions in abdominal emergencies, most frameworks provide limited guidance on incorporating laboratory parameters into diagnostic reasoning or escalation pathways [14,15,16].

This pattern reflects a broader separation between radiology and laboratory medicine in both clinical practice and academic communication. While clinical guidelines advocate multimodal diagnostic reasoning—particularly in high-risk abdominal conditions—radiology publications rarely examine how laboratory information is represented, contextualized, or operationalized within imaging-centered studies [17,18]. As a result, the extent to which laboratory data function as imaging triggers, diagnostic modifiers, or decision-support elements in acute abdominal emergency radiology remains insufficiently characterized.

Methodological content analysis offers a structured approach to examining this gap by systematically evaluating how specific types of information are presented within published literature. Unlike narrative or systematic reviews, content analysis enables quantitative assessment of reporting patterns and contextual roles across a defined body of publications [19,20]. Although this approach has been applied to study reporting quality and decision-support elements in radiology research, its use in examining laboratory–imaging integration in acute abdominal emergencies has been limited.

Understanding how laboratory data are represented and utilized in this context is particularly relevant given the diagnostic complexity and high stakes associated with abdominal emergencies. Biochemical profiles may change rapidly in inflammatory and ischemic conditions, and variability in laboratory interpretation can influence how imaging findings are understood in evolving clinical scenarios [21,22]. Clarifying current reporting practices may therefore help identify patterns, gaps, and opportunities for improving interdisciplinary diagnostic communication.

Accordingly, the aim of this study was to perform a methodological content analysis of published radiology research on acute abdominal emergencies, with emphasis on the representation and utilization of laboratory data. By systematically evaluating how laboratory parameters are reported and integrated within imaging-centered studies, this work seeks to provide objective insight into current reporting practices and to inform future efforts toward more coherent multimodal diagnostic communication in emergency abdominal imaging.

## 2. Materials and Methods

### 2.1. Study Design

This study was designed as a methodological content analysis to systematically examine the representation and utilization of laboratory data in radiology publications addressing acute abdominal emergencies. The methodological approach focused on evaluating how laboratory parameters are reported, in which diagnostic contexts they are used, and whether they actively contribute to imaging-related decision-making within imaging-centered research articles.

Rather than assessing clinical outcomes or diagnostic accuracy, the study specifically targeted the structure and function of reported information within published literature. The unit of analysis was the individual research article, not patients, clinicians, or institutions. Accordingly, the analysis concentrated on textual and methodological elements, including the presence, type, and contextual role of laboratory parameters in relation to CT-based imaging findings.

The study was intentionally limited to original research articles in order to capture real-world reporting practices in imaging-focused scientific communication. Reviews, meta-analyses, and guideline documents were excluded, as they follow fundamentally different reporting conventions and do not reflect primary radiology research workflows. By focusing on original studies, the design aimed to identify patterns of laboratory data integration that emerge during the presentation of imaging results and diagnostic interpretations.

Acute abdominal emergencies were selected as the clinical domain of interest due to their high diagnostic complexity and dynamic laboratory variability, which often directly influence imaging indication, urgency, and interpretation. In these conditions, laboratory markers frequently evolve over short time intervals and may alter both the selection of imaging modality and the diagnostic confidence associated with CT findings. This characteristic makes acute abdominal emergencies particularly suitable for investigating laboratory–imaging interaction within radiology publications.

Importantly, the study did not involve any form of human participation, patient-level information, expert opinion, surveys, or educational assessments. All data were derived exclusively from previously published scientific literature, ensuring that the research remained strictly methodological in nature. Consequently, the study design did not require ethical committee approval and complied with standard ethical principles governing research based on publicly available data sources.

Although this study does not constitute a systematic or scoping review, principles from structured reporting frameworks for evidence synthesis (e.g., PRISMA-ScR) were considered in organizing the study design and reporting structure to enhance methodological transparency.

### 2.2. Data Source and Literature Selection

A comprehensive literature search was conducted using the PubMed/MEDLINE database to identify radiology-focused original research articles addressing acute abdominal emergencies. PubMed was selected as the primary data source due to its broad coverage of peer-reviewed biomedical and radiology journals and its standardized indexing system, which allows reproducible and transparent retrieval of publications.

The search strategy was designed to capture a representative sample of the contemporary radiology literature rather than to exhaustively identify all available studies. Accordingly, the search was restricted to articles published between January 2020 and December 2024, reflecting current imaging practices, reporting standards, and diagnostic paradigms in emergency abdominal radiology. Only articles published in English were included to ensure consistency in content evaluation and terminology.

Search terms were constructed using combinations of Medical Subject Headings (MeSH) and free-text keywords related to acute abdominal emergencies and CT-based imaging. Core concepts included emergency abdominal conditions (e.g., appendicitis, pancreatitis, bowel obstruction, perforation, diverticulitis, mesenteric ischemia) and radiological evaluation using computed tomography. Boolean operators were applied to combine condition-specific terms with imaging-related keywords, and search filters were used to limit results to original research articles.

Given the central role of computed tomography in the evaluation of acute abdominal emergencies, eligibility was limited to studies in which CT constituted the primary imaging modality for diagnostic assessment. Articles focusing exclusively on other imaging techniques—such as ultrasound-only or MRI-only studies—were excluded to maintain methodological homogeneity and to ensure comparability across included publications.

The scope of acute abdominal emergencies encompassed conditions commonly encountered in emergency departments and known to demonstrate substantial laboratory variability, including:

- acute appendicitis
- acute pancreatitis
- bowel obstruction
- perforated viscus
- acute cholecystitis
- diverticulitis
- acute mesenteric ischemia

Publications addressing mixed emergency populations were eligible for inclusion provided that acute abdominal conditions formed a clearly defined component of the study and that CT-based imaging findings were reported in conjunction with clinical context. Screening and coding were performed at the title and abstract level.

Importantly, the literature selection process was not intended to function as a systematic review or meta-analysis. No attempt was made to assess study quality, risk of bias, or clinical effectiveness. Instead, the selection strategy was purposefully designed to assemble a methodologically appropriate corpus of imaging-centered research articles suitable for quantitative and qualitative analysis of reporting practices related to laboratory data usage.

Reproducible Search Strategy.

The PubMed/MEDLINE search was conducted on 15 December 2025. The exact search string used was:

(“acute abdomen” OR appendicitis OR pancreatitis OR “bowel obstruction” OR diverticulitis OR “mesenteric ischemia” OR “perforated viscus” OR cholecystitis) AND (“computed tomography” OR CT) AND (radiology).

Filters applied in PubMed were as follows: language restricted to English; publication date range limited to January 2020 through December 2024; and article type limited to Journal Article to capture original research publications. No full-text restriction was applied at the search stage. The search strategy was designed to identify radiology-centered studies addressing CT-based evaluation of acute abdominal emergency conditions while ensuring reproducibility and transparency of literature retrieval.

### 2.3. Inclusion and Exclusion Criteria

Articles were eligible for inclusion if they met the following criteria: (1) publication in a peer-reviewed journal indexed in the PubMed/MEDLINE database; (2) focus on acute abdominal emergency conditions; (3) radiology-centered content with computed tomography (CT) as a primary imaging modality; (4) original research design; and (5) availability of sufficient information at the title and abstract level to allow assessment of laboratory data representation and contextual role within imaging-centered reporting.

Articles were excluded if they were review articles, meta-analyses, editorials, guidelines, letters, or case reports. Studies focusing exclusively on pediatric populations, non-acute abdominal conditions, or imaging modalities other than CT (e.g., ultrasound- or MRI-only studies) were also excluded. Publications that lacked adequate abstract-level detail to support content analysis of laboratory data utilization were not considered eligible.

### 2.4. Article Screening and Selection Process

The article screening and selection process was conducted using a structured, stepwise approach to ensure methodological transparency and reproducibility. All records retrieved from the PubMed/MEDLINE database search were initially compiled into a single dataset, and duplicate entries were identified and removed prior to screening. The remaining records were then evaluated through a two-stage screening process performed exclusively at the title and abstract level, in accordance with the predefined inclusion and exclusion criteria.

In the first stage, article titles and abstracts were screened to exclude publications that were clearly outside the scope of the study. Records were excluded if they did not address acute abdominal emergency conditions, were not radiology-centered, did not involve CT-based imaging, or represented non-original publication types such as reviews, editorials, guidelines, or case reports. This step was intended to eliminate studies that were thematically or methodologically incompatible with the objectives of the analysis.

In the second stage, the remaining abstracts were assessed in greater detail to confirm eligibility. During this phase, abstracts were examined to verify relevance to acute abdominal emergency presentations and to identify references to laboratory parameters. Particular attention was paid to whether laboratory data were described as background clinical information, imaging triggers, diagnostic modifiers, or elements of integrated diagnostic reasoning. Articles lacking sufficient abstract-level information to support these assessments were excluded.

Because full-text access is not uniformly available for all publications indexed in PubMed, screening and coding were intentionally restricted to the title and abstract level to ensure methodological consistency across the dataset. No full-text–based eligibility assessment, quality appraisal, or risk-of-bias evaluation was performed.

**Figure 1:**
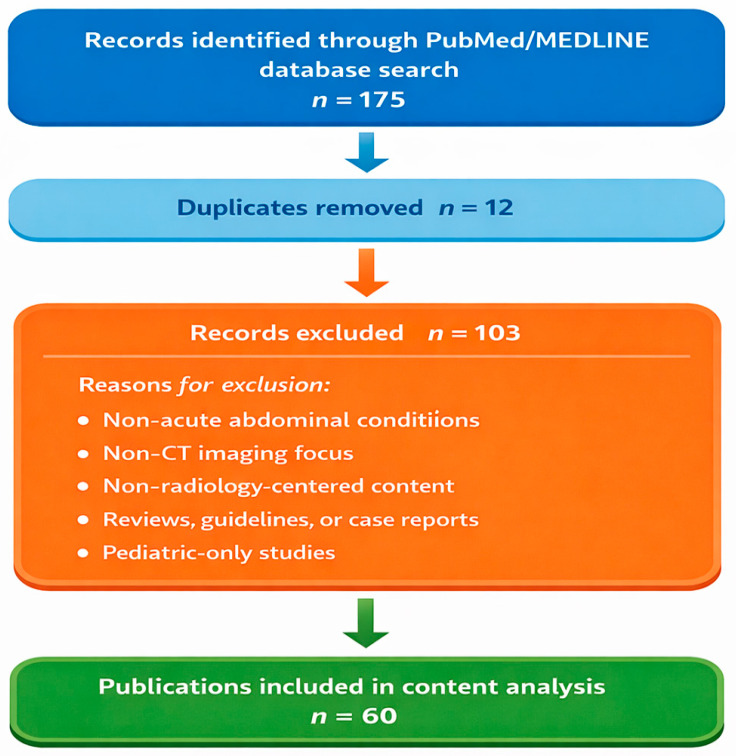
Flow diagram of the study selection process for CT-based radiology publications addressing acute abdominal emergencies.

The screening process followed a structured workflow. After retrieval of records from PubMed, duplicates were removed. Remaining records underwent title and abstract screening to exclude clearly ineligible studies. Articles passing the initial screening were assessed again at the abstract level to confirm relevance to CT-based evaluation of acute abdominal emergencies and to identify laboratory data representation. Studies not meeting inclusion criteria were excluded at this stage. The final dataset consisted of 72 articles included for content analysis. The selection pathway is illustrated in Figure 1.

Because full-text access is not uniformly available for all PubMed-indexed publications and to maintain methodological consistency across the dataset, eligibility assessment and coding were performed at the title and abstract level. This approach reflects the information layer that is consistently accessible during literature retrieval. However, this design choice may limit the capture of laboratory–imaging integration that is described only within full-text sections.

The final dataset was defined based on feasibility and the conceptual objective of identifying reporting patterns rather than estimating prevalence or effect sizes. Accordingly, the analyzed corpus is presented as a methodologically assembled set of publications suitable for pattern characterization rather than as a statistically representative sample.

### 2.5. Data Extraction and Coding Framework

Data extraction and coding were performed using a predefined content analysis framework applied at the title and abstract level. For each included publication, abstract-level information was systematically reviewed to extract variables related to laboratory data reporting and utilization within imaging-centered narratives.

The extracted variables included: (1) presence or absence of laboratory data; (2) types of laboratory parameters mentioned (e.g., inflammatory markers, organ-specific tests); (3) contextual role of laboratory data (background clinical information, imaging trigger, diagnostic modifier, or prognostic indicator); (4) degree of explicit laboratory–imaging integration; and (5) presence of decision-oriented reporting elements. Coding categories were defined a priori to minimize interpretive variability and to enhance internal consistency across the dataset.

This abstract-level coding approach was selected to reflect the information that is uniformly accessible within the PubMed database and to allow systematic comparison of reporting practices across a broad range of radiology publications.

To ensure methodological transparency and reproducibility, the coding process was guided by a structured operational framework developed prior to analysis. Each coding category was defined using explicit criteria to minimize interpretive ambiguity and to standardize classification across all included publications. Decision rules were established to determine when laboratory data were considered merely descriptive versus functionally integrated into imaging-related diagnostic reasoning. Examples reflecting typical abstract-level statements were also identified to illustrate practical application of each category. The operational codebook used in this study is presented below in Table 1.

#### 2.5.1. Coding Reliability Assessment

To assess the reliability of the coding framework, a structured intra-observer reliability check was performed. A randomly selected subset of 15 articles (approximately 20% of the dataset) was re-coded in a blinded manner after a four-week washout period. The second coding session was conducted without reference to the initial coding outcomes. For each article, agreement between the two coding rounds was evaluated across all categorical variables (presence of laboratory data, contextual role, integration status, and decision-oriented reporting). Percent agreement was calculated as the proportion of identical coding decisions divided by the total number of coding decisions. The overall agreement rate was 91%, indicating high consistency in the application of coding definitions and decision rules.

#### 2.5.2. Coding Structure Clarification

The coding categories describing the contextual roles of laboratory data were applied as non-exclusive tags rather than mutually exclusive or hierarchical classifications. A single publication could therefore receive multiple category labels when laboratory parameters served more than one function within the abstract. For example, laboratory values described as background clinical information could also be coded as diagnostic modifiers or elements of laboratory–imaging integration if explicit interpretive linkage was present elsewhere in the abstract.

To avoid conceptual overlap, the category “Background clinical information” refers specifically to laboratory values presented descriptively without explicit linkage to imaging interpretation or diagnostic reasoning. This definition does not preclude simultaneous assignment of additional integration-related categories when functional linkage is articulated in other parts of the abstract.

### 2.6. Outcome Measures

The outcome measures of this methodological content analysis were predefined to quantitatively and qualitatively characterize how laboratory data are represented and utilized within radiology publications focusing on acute abdominal emergencies. Outcomes were selected to reflect not only the presence of laboratory parameters but also their functional role within imaging-centered diagnostic communication.

The primary outcome measure was the overall frequency of laboratory data representation, defined as the proportion of included radiology publications that reported at least one laboratory parameter in association with CT-based imaging findings. This measure was intended to provide a global overview of how commonly laboratory information is incorporated into imaging-focused research on acute abdominal emergencies.

Several secondary outcome measures were defined to further explore patterns of laboratory data utilization. These included the distribution of specific laboratory parameters—such as leukocyte count, C-reactive protein, serum lactate, amylase and/or lipase, and liver function tests—across different acute abdominal emergency conditions. This analysis aimed to identify which laboratory markers were most frequently reported and whether their usage varied according to the underlying pathology.

Another key secondary outcome was the contextual role of laboratory data, categorized according to whether laboratory parameters were presented as background clinical information, explicitly cited as an indication or trigger for imaging, used as a diagnostic modifier in conjunction with CT findings, or referenced as prognostic or severity-related markers. This outcome allowed differentiation between passive mention of laboratory data and active integration into diagnostic reasoning.

The degree of laboratory–imaging integration constituted an additional outcome measure. Publications were evaluated based on whether laboratory findings were explicitly correlated with imaging features within the diagnostic narrative, mentioned without interpretive linkage, or entirely absent from the discussion of imaging results. This measure was designed to assess the depth of interdisciplinary integration within radiology research reporting.

Finally, the presence of decision-oriented reporting was assessed as an outcome measure. Decision-oriented reporting was defined as the inclusion of explicit diagnostic conclusions, escalation statements, or management-related implications that referenced imaging findings with or without accompanying laboratory data. This outcome provided insight into whether laboratory information contributed to actionable diagnostic communication rather than remaining descriptive or ancillary.

Together, these outcome measures enabled a structured assessment of both the quantitative prevalence and qualitative utilization of laboratory data in acute abdominal emergency radiology publications, forming the basis for the descriptive analyses presented in the Results section.

### 2.7. Statistical Analysis

Statistical analysis was conducted to summarize and describe patterns of laboratory data representation and utilization within radiology publications focusing on acute abdominal emergencies. Given the methodological and exploratory nature of the study, analyses were intentionally descriptive, with no hypothesis testing or inferential statistical comparisons planned.

Categorical variables were summarized using absolute frequencies and percentages. These variables included the presence or absence of laboratory data, the type of laboratory parameters reported, the contextual role of laboratory information, the degree of laboratory–imaging integration, and the presence of decision-oriented reporting elements. Descriptive statistics were selected to provide a clear and interpretable overview of reporting practices across the analyzed literature.

To explore variation across different acute abdominal emergency conditions, descriptive distributions were calculated separately for each major diagnostic category, including acute appendicitis, acute pancreatitis, bowel obstruction, perforated viscus, diverticulitis, acute cholecystitis, and acute mesenteric ischemia. This stratified analysis allowed assessment of whether certain laboratory parameters or reporting patterns were preferentially associated with specific emergency conditions, without implying causal or predictive relationships.

No formal inferential statistical tests (such as chi-square testing or regression analysis) were performed, as the primary objective of the study was not to compare groups statistically or to establish associations between variables. Instead, the focus was placed on identifying reporting trends, frequencies, and contextual patterns within contemporary radiology research. This approach aligns with established methodological standards for content analysis studies, in which descriptive statistics are used to characterize informational structures rather than to test clinical hypotheses.

All extracted data were entered into a dedicated database prior to analysis. Statistical analyses were performed using SPSS software version 29 (IBM Corp., Armonk, NY, USA). Data were checked for internal consistency prior to analysis, and summary outputs were reviewed to ensure alignment with the predefined coding framework.

### 2.8. Ethical Considerations

This study was based exclusively on the analysis of previously published scientific literature and did not involve human participants, patient-level data, biological samples, expert opinions, surveys, or any form of direct or indirect human involvement. All data analyzed were obtained from publicly accessible sources.

Accordingly, approval from an institutional review board or ethics committee was not required. The study was conducted in accordance with general ethical principles for research involving publicly available data and complied with relevant international research ethics standards.

## 3. Results

The results of this study consist of the structured outputs of the proposed reporting framework. Unless otherwise specified, percentages are calculated using the total number of included studies (n = 72). Analyses restricted to studies reporting laboratory data are explicitly labeled.

### 3.1. Study Selection and Dataset Characteristics

The database search yielded a pool of radiology publications addressing computed tomography–based evaluation of acute abdominal emergencies. After removal of duplicate records, the remaining articles underwent title and abstract screening according to the predefined inclusion and exclusion criteria. Publications that did not meet the eligibility requirements were excluded during this screening process.

Following completion of the title–abstract screening process, a final dataset of 72 radiology research articles focusing on CT-based evaluation of acute abdominal emergencies was included for content analysis. The characteristics of the included publications and the distribution of laboratory data utilization patterns identified through abstract-level analysis are summarized in Table 2.

Figure 1 shows a flow diagram illustrating the article selection process. Records were identified through the PubMed/MEDLINE database and screened at the title and abstract level according to predefined inclusion and exclusion criteria. The final dataset comprised 72 radiology research articles focusing on CT-based evaluation of acute abdominal emergencies.

The diagram summarizes database retrieval, duplicate removal, title/abstract screening, and final inclusion steps.

### 3.2. Overall Representation of Laboratory Data

Across the final dataset of 72 included radiology publications, laboratory data were reported in a substantial proportion of studies, although the degree of representation varied widely among acute abdominal emergency conditions. Overall, at least one laboratory parameter was mentioned in 61.1% of the analyzed articles, while the remaining 38.9% presented CT-based imaging findings without explicit reference to laboratory or biochemical information.

Among publications that included laboratory data (n = 44), the number and types of reported laboratory parameters varied across studies. Some studies provided only a single laboratory marker as part of the baseline clinical description, whereas others reported multiple biochemical parameters alongside imaging findings. However, even in articles with more extensive laboratory reporting, the depth of integration between laboratory data and imaging interpretation varied substantially.

When stratified by acute abdominal emergency category, differences in laboratory data representation became more apparent. Laboratory data reporting was described in publications addressing conditions with well-established biochemical markers, such as acute pancreatitis and acute appendicitis, as well as in publications focusing on pathologies in which imaging findings are often considered diagnostically decisive, including bowel obstruction and perforated viscus. Nevertheless, even within categories characterized by prominent laboratory abnormalities, laboratory parameters were not consistently integrated into imaging-based diagnostic narratives.

In a notable subset of publications, laboratory data were limited to brief mentions within the introductory or methods sections, serving primarily as contextual background rather than as active contributors to diagnostic reasoning. In contrast, a smaller proportion of studies incorporated laboratory abnormalities into the justification for imaging, interpretation of CT findings, or assessment of disease severity.

Overall, these findings indicate that while laboratory data are frequently present in acute abdominal emergency radiology publications, their representation is inconsistent and often limited in scope. The quantitative distribution of laboratory data representation across diagnostic categories and reporting contexts is summarized in Table 1.

### 3.3. Types of Laboratory Parameters Reported

Analysis of the included publications revealed marked variability in the types of laboratory parameters reported in association with CT-based imaging findings for acute abdominal emergencies. Among studies that included laboratory data (n = 44), inflammatory markers were commonly reported across diagnostic categories.

Leukocyte count and C-reactive protein (CRP) were the predominant laboratory markers, collectively accounting for the majority of laboratory references in the analyzed literature. These parameters were most commonly reported in studies focusing on acute appendicitis, diverticulitis, and acute cholecystitis, where inflammatory activity is central to disease pathophysiology. In many publications, leukocyte count and CRP were presented together as part of baseline clinical assessment; however, the extent to which these markers were incorporated into imaging interpretation varied substantially.

Markers specific to particular abdominal emergencies demonstrated more condition-dependent patterns of reporting. Amylase and/or lipase were frequently cited in publications addressing acute pancreatitis, often appearing as defining clinical parameters that contextualized CT findings and disease severity. In contrast, these enzymes were rarely mentioned in studies focusing on non-pancreatic abdominal emergencies.

Serum lactate was reported less frequently overall but showed a distinct distribution pattern, being disproportionately represented in publications related to acute mesenteric ischemia and complicated bowel pathology. In these studies, elevated lactate levels were often mentioned in relation to disease severity or urgency of imaging; however, explicit correlation with CT findings was not consistently provided.

Liver function tests, including transaminases and bilirubin levels, were primarily reported in studies involving hepatobiliary emergencies, particularly acute cholecystitis and biliary obstruction. Other biochemical parameters, such as electrolyte abnormalities, were infrequently mentioned and, when present, were typically described as ancillary clinical information without direct relevance to imaging interpretation.

Overall, while a wide range of laboratory parameters was reported across acute abdominal emergency radiology publications, the selection and emphasis of specific markers were largely condition-dependent. The relative frequencies of reported laboratory parameters across diagnostic categories are summarized in Table 1. The distribution of contextual roles assigned to laboratory data across the included publications is summarized in Table 3.

### 3.4. Contextual Role of Laboratory Data

Evaluation of the contextual role of laboratory data demonstrated substantial variability in how biochemical information was incorporated within radiology publications focusing on acute abdominal emergencies. Among studies that reported laboratory parameters (n = 44), laboratory data were described in different contextual roles, including background clinical information and roles linked to imaging interpretation or decision-oriented reporting.

In a large proportion of publications, laboratory parameters were presented primarily as background clinical information. In these studies, laboratory values were typically described in introductory sections or patient characteristic summaries without explicit reference to their influence on imaging selection, interpretation, or diagnostic conclusions. Laboratory data in this context functioned as descriptive clinical context rather than as integrated diagnostic elements.

A smaller subset of publications explicitly identified laboratory abnormalities as imaging indications or triggers. In these studies, elevated inflammatory markers, abnormal pancreatic enzymes, or increased serum lactate levels were cited as factors prompting CT examination or CT angiography. This role was most frequently observed in publications addressing acute appendicitis, acute pancreatitis, and suspected mesenteric ischemia, where laboratory abnormalities often precede definitive imaging findings.

Laboratory data were also used as diagnostic modifiers in a limited number of publications. In this context, biochemical parameters were employed to support, refine, or contextualize imaging findings, particularly in cases where CT features were equivocal or where disease severity required further stratification. Examples included the use of inflammatory markers to support borderline imaging findings in appendicitis or the incorporation of lactate levels when interpreting CT signs of bowel ischemia.

In contrast, laboratory parameters were infrequently used as prognostic or severity-related markers within imaging-centered publications. When present, prognostic laboratory data were typically referenced in relation to complications, disease progression, or outcome measures rather than as direct contributors to imaging interpretation.

Overall, the distribution of contextual roles indicated that laboratory data were most commonly used in a descriptive capacity, with less frequent utilization as active imaging triggers or diagnostic modifiers. The quantitative distribution of contextual roles assigned to laboratory data is summarized in Table 2.

### 3.5. Laboratory–Imaging Integration

Assessment of laboratory–imaging integration revealed that explicit correlation between biochemical parameters and CT findings was inconsistently observed across radiology publications focusing on acute abdominal emergencies. Among studies reporting laboratory data (n = 44), explicit laboratory–imaging integration was described in a subset of publications, while in others laboratory parameters were mentioned without interpretive linkage or were not incorporated into imaging-focused discussions.

In publications demonstrating explicit laboratory–imaging integration, laboratory abnormalities were directly referenced in conjunction with specific CT features to support diagnostic conclusions, severity stratification, or escalation decisions. Examples of such reporting were described in studies addressing acute mesenteric ischemia and acute pancreatitis, where biochemical markers such as serum lactate or pancreatic enzymes were presented alongside CT findings to contextualize disease severity or diagnostic interpretation.

In contrast, a larger proportion of publications included laboratory data in a parallel but non-integrated manner. In these studies, laboratory parameters were reported as part of the general clinical background but were not directly correlated with imaging findings in the results or discussion sections. Imaging interpretations in this group relied predominantly on morphological CT features, with laboratory data functioning as ancillary information rather than as integrated diagnostic components.

A notable proportion of publications did not demonstrate any form of laboratory–imaging integration. In these articles, imaging findings were presented independently, without reference to laboratory parameters, even when such data were available elsewhere in the manuscript. This pattern was particularly evident in studies where CT findings were considered diagnostically definitive, such as uncomplicated bowel obstruction or perforated viscus.

Overall, the degree of laboratory–imaging integration varied substantially across acute abdominal emergency conditions and publication types. The distribution of integration patterns is summarized in Table 1.

### 3.6. Decision-Oriented Reporting

Evaluation of decision-oriented reporting focused on how radiology publications articulated imaging findings in relation to explicit diagnostic impressions, clinical urgency, or management-related statements. Within the analyzed dataset, most studies presented CT findings in a descriptive format, whereas a smaller subset included clearly stated decision-oriented elements. Overall, decision-oriented reporting was present in 17 of 72 studies (23.6%).

Publications containing decision-oriented reporting typically included explicit diagnostic interpretations, references to disease severity, or statements indicating potential clinical urgency. In some of these studies, laboratory parameters were described alongside imaging findings within the diagnostic narrative. In such instances, laboratory data and CT features were presented together as part of the reported clinical context rather than as isolated pieces of information.

In contrast, many publications described imaging findings without extending interpretation to diagnostic certainty, risk stratification, or management implications. In these articles, laboratory data—when mentioned—were commonly presented as general clinical background and were not incorporated into the imaging-centered conclusions. Imaging results in this group were primarily expressed as morphological observations without explicit linkage to clinical decision statements.

Decision-oriented reporting appeared across different acute abdominal emergency categories and publication types within the dataset. Some studies describing laboratory–imaging integration also included decision-oriented elements, while others did not. These observations represent descriptive reporting patterns and are presented without inferential statistical comparison. The distribution of decision-oriented reporting across the analyzed publications is summarized in Table 1.

A descriptive cross-tabulation of laboratory–imaging integration and decision-oriented reporting is provided to illustrate reporting distributions within the dataset. Among studies describing explicit laboratory–imaging integration (n = 11), decision-oriented reporting was present in 6 studies. Among studies without explicit integration (n = 61), decision-oriented reporting was present in 11 studies. These values are presented as frequency distributions only, and no inferential statistical comparison was performed.

### 3.7. Summary of Key Findings

This methodological content analysis describes how laboratory data are represented within radiology publications addressing acute abdominal emergencies. Laboratory parameters were mentioned in many studies, and their contextual roles differed across diagnostic topics and reporting formats. Inflammatory markers and condition-specific laboratory parameters appeared in various contexts, including background clinical description and diagnostic discussion.

References to laboratory abnormalities as imaging triggers, diagnostic modifiers, or components of decision-oriented statements were identified in a subset of publications. In some studies, laboratory parameters were described together with CT findings within the diagnostic narrative, while in others imaging findings were presented independently of laboratory context.

Decision-oriented reporting was present in part of the analyzed literature. Some publications included explicit diagnostic impressions or statements related to clinical context, whereas others presented imaging findings primarily as morphological descriptions. Laboratory–imaging integration and decision-oriented language were distributed variably across the dataset.

Overall, these observations characterize reporting practices within the analyzed radiology literature. The frequencies of laboratory data representation, contextual roles, integration patterns, and decision-oriented reporting are summarized in Table 1, and the article selection process is illustrated in Figure 1. These findings reflect patterns of research communication and do not imply statistical associations, predictive relationships, or causal effects.

## 4. Discussion

This methodological content analysis offers an in-depth evaluation of how laboratory data are represented and operationalized within radiology publications focusing on acute abdominal emergencies. The principal finding of this study is that although laboratory parameters are frequently mentioned, their systematic and functional integration into imaging-centered diagnostic reasoning remains limited, inconsistent, and highly variable across the contemporary radiology literature.

Acute abdominal emergencies constitute a clinical domain in which diagnostic uncertainty is common and rapid decision-making is essential. In such settings, laboratory data and imaging findings are inherently interdependent. Despite this, the present analysis demonstrates that radiology publications often reflect a fragmented representation of these data sources, with laboratory parameters frequently relegated to background clinical context rather than being incorporated as active diagnostic components.

### 4.1. Presence of Laboratory Data Does Not Equate to Diagnostic Utilization

One of the most salient findings of this study is the clear conceptual and functional distinction between the presence of laboratory data and their actual diagnostic utilization within radiology publications addressing acute abdominal emergencies. Although laboratory parameters are frequently reported, their inclusion does not necessarily translate into meaningful integration within imaging interpretation or diagnostic reasoning [23].

In many analyzed publications, laboratory data were presented as part of baseline clinical characteristics or introductory patient descriptions, often limited to brief mentions of inflammatory markers or disease-specific biochemical abnormalities. These laboratory parameters were typically reported without explicit reference to how they influenced imaging selection, interpretation of CT findings, or diagnostic confidence [24,25]. As a result, laboratory data frequently functioned as descriptive contextual information rather than as active contributors to radiological reasoning.

This pattern reflects a longstanding convention in radiology manuscript structure, in which imaging findings are emphasized as the primary source of diagnostic insight, while clinical and laboratory information is introduced primarily to characterize study populations. Such an approach may be appropriate in scenarios where imaging findings are unequivocal; however, in acute abdominal emergencies—where laboratory values often evolve dynamically and may substantially alter pretest probability—this separation may be methodologically limiting.

The disconnect between reporting and utilization is particularly notable given the central role of laboratory data in real-world emergency diagnostic workflows. In clinical practice, abnormal laboratory findings frequently guide imaging decisions, influence protocol selection, and shape interpretive emphasis. For example, elevated inflammatory markers may increase suspicion for appendicitis or diverticulitis, while rising serum lactate levels may prompt urgent CT angiography in suspected mesenteric ischemia [26,27]. Despite this, such laboratory-driven diagnostic reasoning is often underarticulated in radiology publications.

Moreover, the inclusion of laboratory data without explicit interpretive linkage may inadvertently obscure the diagnostic reasoning process. Readers are left to infer the relevance of biochemical abnormalities rather than being guided through an integrated diagnostic narrative. This limitation may reduce the educational value of imaging-centered research and impede knowledge transfer to trainees and clinicians seeking to understand how laboratory and imaging data are jointly interpreted in emergency settings.

The findings of this study therefore suggest that the mere reporting of laboratory data does not ensure their diagnostic utilization. Instead, explicit articulation of how laboratory parameters interact with imaging findings appears necessary to convey clinically meaningful insights. Without such integration, laboratory data risk being relegated to peripheral information, despite their recognized importance in emergency diagnostic decision-making.

From a research communication perspective, this observation underscores the need for greater transparency in how laboratory data are operationalized within imaging interpretation. More explicit integration of laboratory findings into radiological narratives may better reflect real-world diagnostic processes and enhance the translational relevance of radiology publications addressing acute abdominal emergencies [28].

### 4.2. Condition-Specific Variability in Laboratory–Imaging Relationships

The present analysis identified differences in how laboratory data are represented within radiology publications addressing various acute abdominal emergency conditions. These differences appear in the manner laboratory parameters are described alongside imaging findings and in the contexts in which they are mentioned within diagnostic narratives.

In publications addressing conditions with well-established biochemical correlates, such as acute pancreatitis and acute appendicitis, laboratory parameters were frequently included as part of the reported clinical context. In acute pancreatitis, elevations in amylase and lipase are central to diagnostic definitions and severity frameworks, and these laboratory abnormalities are often described together with CT findings in discussions of disease characteristics and complications [29,30]. Similarly, studies focusing on acute appendicitis commonly referenced inflammatory markers, reflecting their routine role in clinical assessment and imaging referral pathways [31]. In many of these publications, laboratory parameters were presented descriptively, with varying degrees of explicit linkage to imaging interpretation.

In publications addressing conditions where imaging findings are often emphasized in diagnostic discussions, such as bowel obstruction and perforated viscus, laboratory data were described less prominently within imaging-centered narratives. In these studies, CT features—including bowel dilation, transition points, extraluminal air, or contrast leakage—formed the primary focus of reported findings, while laboratory parameters, when mentioned, were typically included as general clinical information rather than as components of imaging interpretation [32,33,34].

Publications concerning clinically complex and time-sensitive conditions, including acute mesenteric ischemia, also described laboratory parameters such as serum lactate in conjunction with imaging findings. In these contexts, laboratory abnormalities were included as part of the broader clinical description accompanying CT-based evaluation, reflecting established clinical considerations related to disease severity and systemic effects [35].

Overall, the observed differences indicate that laboratory–imaging relationships are described in varying ways across radiology publications focusing on different acute abdominal emergency conditions. These observations characterize reporting patterns within the analyzed literature and do not imply statistical associations, predictive relationships, or causal effects.

### 4.3. Laboratory Data as Imaging Triggers

Laboratory data are frequently described in clinical literature as factors considered during imaging decision processes in acute abdominal emergencies. Biomarkers such as leukocyte count, C-reactive protein, pancreatic enzymes, and serum lactate are commonly referenced in clinical contexts related to imaging selection, protocol considerations, and assessment of urgency [36].

Clinical sources note that elevated inflammatory markers may be present in suspected acute appendicitis, diverticulitis, or complicated cholecystitis, and that abnormal pancreatic enzymes are described in association with suspected acute pancreatitis. Similarly, increased serum lactate levels are discussed in relation to suspected mesenteric or bowel ischemia and may be mentioned in contexts involving CT angiography [37,38]. These laboratory parameters are included in clinical descriptions of diagnostic workflows for acute abdominal emergencies.

Within the analyzed radiology publications, references to laboratory data as imaging triggers were present in some studies. In these cases, laboratory abnormalities were mentioned in proximity to descriptions of imaging indications; however, the extent of explanation regarding how laboratory findings influenced imaging selection or urgency varied across reports. In several publications, laboratory values appeared as part of general clinical descriptions, while the rationale for imaging was not elaborated in detail.

Radiology manuscripts follow diverse reporting conventions, and the description of imaging indications is not uniform across publications. In some articles, indications for CT are summarized briefly, while in others they are described in greater detail. This variation is also reflected in how laboratory findings are presented in relation to imaging decisions [39].

Overall, the analyzed literature demonstrates differences in how laboratory data are described in connection with imaging indications. These observations pertain to reporting practices within radiology publications and do not represent direct evaluation of clinical workflows or decision-making processes.

### 4.4. Laboratory–Imaging Integration and Decision-Oriented Reporting

This analysis examined how laboratory–imaging integration and decision-oriented reporting were described within radiology publications addressing acute abdominal emergencies. Across the analyzed literature, imaging findings were commonly presented in a descriptive format, while explicit diagnostic conclusions, urgency statements, or management-related expressions were reported in a smaller subset of studies. Publications varied in how imaging findings, laboratory data, and clinical interpretation were articulated together within the reporting structure [40,41].

In studies describing laboratory–imaging integration, laboratory parameters and CT findings were presented together within the diagnostic narrative. In these reports, laboratory abnormalities were discussed alongside imaging features when outlining disease characteristics, severity, or clinical context. Examples of this reporting style were described in publications addressing a range of acute abdominal emergency conditions, including clinically complex and time-sensitive scenarios such as acute mesenteric ischemia, severe acute pancreatitis, and complicated intra-abdominal infections [42,43].

In other publications, imaging findings were presented without explicit integration of laboratory data. In these instances, laboratory parameters—when mentioned—were typically included as general clinical background rather than as components of imaging interpretation. Imaging descriptions in this group primarily focused on morphological CT findings, with limited extension toward diagnostic or management-oriented statements. This style of reporting has been noted in discussions of radiology communication practices and interpretive boundaries in emergency imaging contexts [44].

Decision-oriented reporting was not uniformly present across the dataset. Some publications included explicit diagnostic impressions, references to clinical urgency, or statements related to patient management, whereas others limited reporting to descriptive imaging observations. Differences in reporting style have been discussed in the context of radiology communication and structured reporting frameworks, which address variability in how diagnostic information is conveyed in imaging literature [45].

From an educational perspective, the limited presence of decision-oriented language may be relevant for how imaging research is used as a learning resource. Radiology trainees and early-career clinicians often rely on published literature not only to recognize imaging patterns but also to understand how imaging findings are expressed within diagnostic narratives. Variability in reporting approaches may therefore influence how imaging interpretation is contextualized within clinical reasoning.

The findings of this study also reflect broader discussions regarding communication between radiology research and clinical decision-making frameworks. Clinical guidelines for acute abdominal emergencies emphasize the integration of laboratory results, imaging findings, and clinical presentation when guiding management decisions; however, the manner in which this integration is described in radiology publications is not uniform [46,47].

Overall, the observations presented here describe patterns in how laboratory data, imaging findings, and decision-oriented language are represented within the analyzed literature. These patterns are reported descriptively and do not imply statistical associations, causal relationships, or predictive effects. Instead, they illustrate variations in reporting approaches within radiology publications focusing on acute abdominal emergencies.

### 4.5. Implications for Radiology Research Communication

The findings of this study have important implications for how radiology research is conceptualized, structured, and communicated, particularly in the context of acute abdominal emergencies. Although contemporary emergency medicine increasingly emphasizes multimodal diagnostic reasoning, radiology publications continue to reflect a predominantly imaging-centric communication model, in which laboratory data are inconsistently integrated into diagnostic narratives [48].

Radiology research plays a dual role: it advances scientific knowledge while simultaneously serving as an educational and communicative bridge between imaging specialists and clinicians. When laboratory data are presented without explicit integration into imaging interpretation, this bridge may be weakened. Readers are often provided with detailed morphological descriptions but limited insight into how imaging findings interact with laboratory abnormalities to inform diagnostic confidence, urgency, or management decisions [49].

The observed heterogeneity in laboratory–imaging integration suggests the absence of a shared reporting paradigm within radiology research. While some publications provide integrated, decision-oriented narratives, others adhere to traditional descriptive frameworks that isolate imaging findings from broader clinical context. This inconsistency may hinder interdisciplinary communication, particularly in emergency settings where radiologists, emergency physicians, and surgeons rely on shared diagnostic language to guide rapid decision-making [50].

From a research dissemination perspective, the limited articulation of laboratory–imaging relationships may also affect the interpretability and reproducibility of imaging studies. Without clear documentation of how laboratory data influenced imaging interpretation or diagnostic reasoning, it becomes difficult for readers to fully contextualize study findings or apply them to different clinical environments. This limitation may be particularly relevant for multicenter studies or international readerships, where diagnostic thresholds and workflows may vary.

Structured reporting frameworks and standardized manuscript templates have been proposed as potential solutions to improve clarity and consistency in radiology communication. By explicitly defining the role of laboratory parameters within imaging narratives, such frameworks may encourage more systematic integration of biochemical data into radiological interpretation. This approach may be especially valuable in acute abdominal emergencies, where diagnostic uncertainty is common and interdisciplinary collaboration is essential [51].

Importantly, improved laboratory–imaging integration in radiology publications does not imply encroachment upon clinical decision-making responsibilities. Rather, it represents a more transparent articulation of diagnostic reasoning processes that already occur in practice. Explicitly describing how laboratory data contextualize imaging findings may enhance the educational value of radiology research and better align published studies with real-world diagnostic workflows [52].

Finally, the implications of these findings extend beyond emergency radiology. As artificial intelligence, structured reporting, and decision-support systems become increasingly integrated into imaging practice, clear documentation of how laboratory and imaging data interact will be critical. Radiology research that fails to articulate these relationships may limit the development and validation of multimodal diagnostic tools that reflect the complexity of contemporary clinical decision-making [53].

### 4.6. Methodological Considerations

From a methodological standpoint, this study adopts a content analysis framework to examine reporting practices within the radiology literature rather than to evaluate diagnostic accuracy, clinical outcomes, or therapeutic effectiveness. This distinction is fundamental to the interpretation of the findings. Content analysis enables systematic examination of how information is structured, contextualized, and operationalized in scientific communication, offering insights that are not readily accessible through conventional outcome-based or accuracy-focused study designs [54,55].

Unlike systematic reviews or meta-analyses, the present study does not aim to synthesize evidence or compare diagnostic performance across studies. Instead, it focuses on the unit of analysis as the published article itself, evaluating how laboratory data are represented and utilized within imaging-centered narratives. This approach allows identification of implicit norms, recurring patterns, and communicative gaps in radiology research that may otherwise remain unrecognized.

The decision to apply descriptive rather than inferential statistical methods reflects the exploratory nature of the study and the characteristics of the analyzed dataset. Because the included publications do not represent a random sample and because the primary outcomes relate to reporting practices rather than independent clinical observations, inferential statistical testing would be methodologically inappropriate and potentially misleading. Emphasis on frequencies, proportions, and qualitative contextual classification is therefore consistent with established principles of content analysis methodology.

Another important methodological consideration is the use of a predefined coding framework. By establishing coding categories prior to analysis, the study minimizes interpretive drift and enhances internal consistency. The framework was designed to capture both the presence of laboratory data and their functional role within imaging interpretation, allowing differentiation between passive reporting and active diagnostic integration. Such structured coding approaches are widely recommended in content analysis research to improve transparency and reproducibility [56].

The use of a single-investigator coding process represents both a strength and a limitation. On one hand, single-coder analysis ensures consistency in the application of coding rules across the dataset, reducing variability introduced by differing interpretive thresholds. On the other hand, the absence of interobserver agreement analysis limits assessment of coding reliability. However, given the clearly defined coding categories and the objective nature of many classification decisions (e.g., presence or absence of laboratory data), this limitation is mitigated to some extent [57].

The selection of a representative rather than exhaustive sample of publications is another methodological feature that warrants consideration. The study was designed to capture prevailing reporting practices within the contemporary radiology literature rather than to include every eligible publication. This approach prioritizes analytical depth and feasibility over completeness and is consistent with exploratory methodological research aimed at identifying patterns rather than estimating population parameters [58].

Finally, it is important to emphasize that the findings of this study should be interpreted as reflections of reporting behavior, not as direct representations of clinical practice. Radiologists and clinicians may integrate laboratory and imaging data extensively in real-world settings, even if such integration is not explicitly articulated in published manuscripts. Consequently, the observed patterns highlight communicative tendencies within radiology research rather than deficiencies in clinical diagnostic reasoning [59].

### 4.7. Limitations

Several limitations should be considered when interpreting the findings. The analysis was restricted to English-language radiology publications indexed in a single database and focused exclusively on CT-based studies. While this ensured methodological consistency, it may limit generalizability to other languages, databases, or imaging modalities with different diagnostic workflows and reporting conventions [60,61].

The study evaluated reporting practices rather than real-world clinical behavior. Laboratory data may be integrated extensively in clinical decision-making even when not explicitly documented in manuscripts; thus, the findings reflect communication patterns within the literature rather than clinical diagnostic performance.

Although a predefined coding framework and decision rules were used to enhance consistency, content analysis inherently involves interpretive judgment. Subtle nuances in narrative reasoning may not be fully captured by categorical classification.

Because eligibility assessment and coding were performed at the title and abstract level, laboratory–imaging integration occurring only within full texts may not have been captured. This methodological choice may therefore lead to underestimation of integration depth and decision-oriented reporting.

A key methodological limitation of this study is that the unit of analysis was restricted to title and abstract content rather than full-text articles. While this approach ensured standardization and reproducibility across a broad range of publications, it may have led to underrepresentation of laboratory–imaging integration occurring only in full-text discussions, methods sections, or results narratives. Consequently, the reported frequencies of integration depth and decision-oriented reporting likely represent conservative estimates rather than the full extent of interdisciplinary diagnostic reasoning present in the original studies.

Although coding was conducted by a single investigator, a structured blinded recoding procedure demonstrated high intra-observer agreement, supporting the internal consistency of the applied coding framework.

### 4.8. Suggested Reporting Checklist for Laboratory–Imaging Integration

Based on the reporting patterns identified in this analysis, a concise set of practical recommendations is proposed to enhance laboratory–imaging integration and decision-oriented reporting in radiology-focused publications (Table 4).

This checklist is intended as a practical guidance tool rather than a prescriptive reporting requirement.

### 4.9. Comparison with Existing Literature and Contribution to the Field

When compared with the existing radiology literature, the findings of this study highlight an important and previously underexplored discrepancy between acknowledged clinical practice and academic reporting behavior. Prior studies and clinical guidelines consistently emphasize the integrated use of laboratory data, imaging findings, and clinical presentation in the evaluation of acute abdominal emergencies. In contrast, the present analysis demonstrates that such multimodal diagnostic reasoning is not consistently reflected in radiology research publications.

Previous radiology-focused studies have primarily concentrated on imaging morphology, diagnostic accuracy, and complication detection, often treating laboratory parameters as ancillary clinical descriptors rather than as integral components of diagnostic reasoning. While this imaging-centric approach has contributed substantially to advances in emergency radiology, it has also resulted in limited exploration of how laboratory data actively influence imaging interpretation, diagnostic confidence, and decision-making processes within published research.

Existing literature addressing structured reporting and radiology communication has highlighted the importance of clarity, consistency, and clinical relevance in imaging reports. However, these discussions have largely focused on imaging terminology and report structure rather than on the content-level integration of laboratory data. The present study extends this body of work by systematically examining not only whether laboratory data are mentioned but also how they are functionally utilized within imaging-centered narratives.

Importantly, this study differs from prior work by adopting a methodological content analysis approach, allowing evaluation of reporting practices across a broad spectrum of acute abdominal emergency conditions. Rather than assessing diagnostic performance or outcomes, the analysis focuses on communication patterns and implicit reporting norms within the radiology literature. This perspective provides novel insight into how radiology research conveys diagnostic reasoning and where gaps between clinical practice and academic communication may exist.

The principal contribution of this study to the field lies in its identification of laboratory–imaging integration as a determinant of decision-oriented reporting. By demonstrating that publications with explicit laboratory–imaging integration are more likely to include actionable diagnostic conclusions, this work underscores the potential value of integrated reporting for enhancing the clinical relevance of radiology research. This contribution is particularly timely given the increasing emphasis on interdisciplinary collaboration, structured reporting, and the development of multimodal decision-support systems in radiology.

In summary, while existing literature has extensively addressed the technical and morphological aspects of emergency imaging, the present study contributes a novel communicative and methodological perspective, highlighting how laboratory data are underutilized in radiology publications despite their central role in emergency diagnostic workflows. These findings provide a foundation for future efforts aimed at improving research communication standards and aligning radiology publications more closely with real-world clinical reasoning.

## 5. Conclusions

This methodological content analysis indicates that laboratory data are commonly mentioned in radiology publications addressing acute abdominal emergencies; however, the manner in which these data are incorporated into imaging-centered reporting differs across studies. Laboratory parameters are described in various contextual roles, including background clinical information, imaging triggers, diagnostic modifiers, and elements appearing within decision-oriented statements.

The analysis further shows that laboratory–imaging integration is not uniformly articulated within the examined literature. Some publications present laboratory data and imaging findings together within the diagnostic narrative, whereas others describe imaging findings independently of laboratory context. These differences reflect variations in reporting approaches rather than measured associations between variables.

Decision-oriented reporting is also variably represented. In some studies, imaging findings are accompanied by explicit diagnostic impressions or references to clinical context, while in others imaging descriptions remain primarily morphological. The observed patterns characterize how information is expressed within published radiology research and do not imply statistical relationships or causal effects.

Importantly, the findings of this study pertain to reporting practices within CT-focused, English-language publications indexed in PubMed and evaluated at the title–abstract level. They should therefore be interpreted as a description of research communication patterns rather than as a reflection of real-world diagnostic workflows.

Overall, the results highlight differences in how laboratory data, imaging findings, and clinical interpretation are articulated in the radiology research literature. These observations may inform future discussions on reporting clarity and communication practices within emergency abdominal imaging.

## Figures and Tables

**Table 1:** Operational Coding Framework Used in the Study.

Category	Operational Definition	Decision Rule	Example from an Abstract
Background Clinical Information	Laboratory values mentioned only as general patient description without influence on imaging interpretation	If lab value is stated but not linked to imaging decision or diagnosis	“Patients presented with abdominal pain and elevated CRP levels.”
Imaging Trigger	Laboratory abnormality explicitly cited as a reason for performing CT	Lab value precedes or justifies imaging	“CT was performed due to elevated serum lactate levels and suspicion of mesenteric ischemia.”
Diagnostic Modifier	Laboratory data used to support, refine, or strengthen imaging-based diagnosis	Lab value alters diagnostic confidence	“Borderline CT findings combined with leukocytosis supported the diagnosis of appendicitis.”
Prognostic Indicator	Laboratory parameter linked to disease severity, outcome, or complication risk	Lab value referenced in severity context	“High CRP levels were associated with severe pancreatitis.”
Explicit Laboratory–Imaging Integration	Laboratory and CT findings discussed together within diagnostic reasoning	Direct interpretive linkage present	“Elevated lactate levels in conjunction with bowel wall hypoenhancement on CT suggested ischemia.”
Decision-Oriented Reporting	Abstract contains explicit diagnostic conclusion, urgency, or management implication	Imaging findings lead to action statement	“Findings warrant urgent surgical evaluation.”
No Laboratory Data	No laboratory parameter mentioned	No biochemical reference in abstract	“CT findings demonstrated…” (without lab mention)

**Table 2:** Characteristics of Included Publications and Laboratory Data Utilization. (Title–abstract level content analysis, n = 72).

Variable	n (%)
Total included publications	72 (100%)
Primary imaging modality	
–Computed tomography (CT)	72 (100%)
Acute abdominal emergency category	
–Appendicitis-related conditions	18 (25.0%)
–Acute pancreatitis	9 (12.5%)
–Biliary emergencies	8 (11.1%)
–Bowel obstruction/ischemia	15 (20.8%)
–Diverticulitis/colonic emergencies	10 (13.9%)
–Non-specific or mixed acute abdominal pain	12 (16.7%)
Laboratory data mentioned	
–Yes	44 (61.1% of all studies, n = 72)
–No	28 (38.9%)
Laboratory data used as imaging trigger	19 (26.4% of all studies, n = 72)
Laboratory data used as diagnostic modifier	14 (19.4% of all studies, n = 72)
Explicit laboratory–imaging integration	11 (15.3% of all studies, n = 72)
Decision-oriented reporting present	17 (23.6% of all studies, n = 72)
Most frequently mentioned laboratory parameters *	
–White blood cell count/leukocytosis	31 (43.1%)
–C-reactive protein (CRP)	26 (36.1%)
–Liver function tests/bilirubin	18 (25.0%)
–Lipase/amylase	11 (15.3%)
–Lactate	7 (9.7%)

* Based on explicit mention in the title or abstract.

**Table 3:** Contextual Roles of Laboratory Data in Imaging-Centered Reporting. (Percentages are calculated based on the total number of included publications (n = 72) unless otherwise specified).

Contextual Role of Laboratory Data	Description	n (%)
Background clinical information	Laboratory values reported as part of general clinical context without direct influence on imaging interpretation	44 (61.1%)
Imaging trigger	Laboratory abnormalities explicitly cited as indications for CT examination	19 (26.4%)
Diagnostic modifier	Laboratory findings used to support, refine, or alter imaging-based diagnosis	14 (19.4%)
Prognostic or severity indicator	Laboratory parameters linked to disease severity, complication risk, or clinical stratification	9 (12.5%)
Explicit laboratory–imaging integration	Laboratory and imaging findings jointly discussed within diagnostic reasoning	11 (15.3%)

Percentages are calculated based on the total number of included publications. Categories are not mutually exclusive.

**Table 4:** Suggested Reporting Checklist for Laboratory–Imaging Integration in Radiology Publications.

Element	Description
Explicit laboratory context	Key laboratory abnormalities relevant to the studied condition should be stated in the abstract when available
Laboratory data as imaging trigger	Clarify whether laboratory findings influenced the decision to perform CT or imaging urgency
Interpretive linkage	Correlate laboratory values with imaging findings when they affect diagnostic reasoning
Diagnostic role clarification	Specify whether laboratory parameters function as supportive evidence, severity markers, or diagnostic modifiers
Decision-oriented statement	Include explicit diagnostic or management-related implications when imaging findings indicate urgency
Severity and risk stratification	Describe relationships between laboratory parameters and imaging indicators of disease severity
Transparency of integration level	Clarify whether laboratory data are central to interpretation or merely descriptive

## Data Availability

The analyzed data consist of published article metadata and narrative content available through the PubMed/MEDLINE database. No original patient-level data were generated.

## References

[1] Kopitnik N.L., Kashyap S., Dominique E. (2025). Acute abdomen. StatPearls.

[2] Cartwright S.L., Knudson M.P. (2008). Evaluation of acute abdominal pain in adults. Am. Fam. Physician.

[3] Rosen M.P., Siewert B., Sands D.Z., Bromberg R., Edlow J., Raptopoulos V. (2003). Value of abdominal CT in the emergency department for patients with abdominal pain. Eur. Radiol..

[4] Coursey C.A., Nelson R.C., Patel M.B., Cochran C., Dodd L.G., DeLong D.M., Beam C.A., Vaslef S. (2010). Making the diagnosis of acute appendicitis: Do more preoperative CT scans mean fewer negative appendectomies?. Radiology.

[5] Leite N.P., Pereira J.M., Cunha R., Pinto P., Sirlin C. (2009). CT evaluation of the acute abdomen: Practical insights. Radiographics.

[6] Andersson R.E. (2004). Meta-analysis of the clinical and laboratory diagnosis of appendicitis. Br. J. Surg..

[7] Schellekens D.H., Hulsewé K.W., van Acker B.A., van Bijnen A.A., de Jaegere T.M., Sastrowijoto S.H., Buurman W.A., Derikx J.P. (2013). Evaluation of the diagnostic accuracy of plasma markers for early diagnosis in patients suspected for acute appendicitis. Acad. Emerg. Med..

[8] van Randen A., Laméris W., van Es H.W., van Heesewijk H.P.M., van Ramshorst B., Hove W.T., Bouma W.H., van Leeuwen M.S., van Keulen E.M., Ten Hove W. (2011). A comparison of the accuracy of ultrasound and computed tomography in common diagnoses causing acute abdominal pain. Eur. Radiol..

[9] Treskes N., Persoon A.M., van Zanten A.R.H. (2017). Diagnostic accuracy of serological biomarkers to detect acute mesenteric ischemia: A systematic review. Intern. Emerg. Med..

[10] Zafirovski A., Zafirovska M., Kuhelj D., Pintar T. (2024). The impact of biomarkers on early detection of acute mesenteric ischemia. Biomedicines.

[11] Bollen T.L. (2012). Imaging of acute pancreatitis: Update of the revised Atlanta classification. Radiol. Clin. N. Am..

[12] Alkasab T.K., Albadawi H., Abujudeh H.H. (2014). Structured reporting in radiology: Current status and future directions. AJR Am. J. Roentgenol..

[13] Brook O.R., Brook A., Vollmer C.M., Kent T.S., Raptopoulos V. (2015). Structured reporting of CT scans for acute abdominal pain: Impact on diagnostic clarity. Emerg. Radiol..

[14] Brady A.P. (2018). Radiology reporting-from Hemingway to HAL?. Insights Imaging.

[15] Bosmans J.M., Weyler J.J., De Schepper A.M., Parizel P.M. (2011). The radiology report as seen by indicate clinicians: Results of a survey. Eur. Radiol..

[16] Sobez L.M., Kim S.H., Angstwurm M., Störmann S., Pförringer D., Schmidutz F., Prezzi D., Kelly-Morland C., Sommer W.H., Sabel B. (2019). Creating high-quality radiology reports in foreign languages through multilingual structured reporting. Eur. Radiol..

[17] Sartelli M., Chichom-Mefire A., Labricciosa F.M., Hardcastle T., Abu-Zidan F.M., Adesunkanmi A.K., Ansaloni L., Bala M., Balogh Z.J., Beltrán M.A. (2020). WSES guidelines for management of acute abdominal emergencies. World J. Emerg. Surg..

[18] Scheirey C.D., Fowler K.J., Therrien J.A., Kim D.H., Al-Refaie W.B.H., Camacho M.A., Cash B.D., Chang K.J., Garcia E.M., Kambadakone A.R. (2018). ACR Appropriateness Criteria® Acute Nonlocalized Abdominal Pain. J. Am. Coll. Radiol..

[19] Krippendorff K. (2013). Content Analysis: An Introduction to Its Methodology.

[20] Neuendorf K.A. (2017). The Content Analysis Guidebook.

[21] Moncy A.A., Kavalakat A.J., Vikraman B. (2023). Utility of serum lactate in identifying ischemia in acute intestinal obstruction. Cureus.

[22] Prathapan A., Walwekar A., Hosamani I.R., Walwekar R. (2024). LDH levels in acute intestinal obstruction as a marker of bowel gangrene. Cureus.

[23] Minten L., Messiaen P., Van der Hilst J. (2022). Acute abdominal pain: A challenging diagnosis. Acta Gastroenterol. Belg..

[24] Atema J.J., Gans S.L., van Randen A., Laméris W., van Es H.W., van Heesewijk J.P., van Ramshorst B., Bouma W.H., Ten Hove W., van Keulen E.M. (2019). Comparison of Imaging Strategies with Conditional versus Immediate CT in Patients with Suspected Acute Appendicitis. Diagnostics.

[25] Sammalkorpi H.E., Leppäniemi A., Mentula P. (2020). Diagnostic Performance of Inflammatory Markers in Acute Appendicitis. Diagnostics.

[26] Gans S.L., Atema J.J., Stoker J., Boermeester M.A. (2021). Guideline-Based Diagnostic Pathways for Acute Abdominal Pain in the Emergency Department. Diagnostics.

[27] Acosta S. (2014). Mesenteric ischemia. Br. J. Surg..

[28] Oldenburg W.A., Lau L.L., Rodenberg T.J., Edmonds H.J., Burger C.D. (2004). Acute mesenteric ischemia: A clinical review. Arch. Intern. Med..

[29] Krajewski S., Brown J., Phang P.T., Raval M., Brown C.J. (2011). Impact of computed tomography of the abdomen on clinical outcomes in patients with acute right lower quadrant pain: A meta-analysis. Can. J. Surg..

[30] Banks P.A., Bollen T.L., Dervenis C., Gooszen H.G., Johnson C.D., Sarr M.G., Tsiotos G.G., Vege S.S. (2013). Acute Pancreatitis Classification Working Group. Classification of acute pancreatitis—2012: Revision of the Atlanta classification. Gut.

[31] Mortelé K.J., Wiesner W., Intriere L., Shankar S., Zou K.H., Kalantari B.N., Perez A., Vansonnenberg E., Ros P.R., Banks P.A. (2004). A modified CT severity index for evaluating acute pancreatitis. AJR Am. J. Roentgenol..

[32] Shogilev D.J., Duus N., Odom S.R., Shapiro N.I. (2014). Diagnosing appendicitis in adults and children. West. J. Emerg. Med..

[33] Sarr M.G. (2011). Management of intestinal obstruction. Surg. Clin. N. Am..

[34] Podda M., Pisanu A., Sartelli M., Coccolini F., Damaskos D., Augustin G., Khan M., Pata F., De Simone B., Ansaloni L. (2021). Diagnosis of acute appendicitis based on clinical scores: Is it a myth or reality?. Acta Biomed..

[35] Park J.H., LOCAT Group (2020). Diagnostic Imaging Utilization and Clinical Outcomes in Suspected Acute Appendicitis. J. Clin. Med..

[36] Kärkkäinen J.M., Lehtimäki T.T., Manninen H., Paajanen H. (2021). Acute Mesenteric Ischemia—Diagnosis and Treatment: A Review. J. Clin. Med..

[37] European Society of Radiology (ESR) (2018). Structured reporting in emergency radiology. Insights Imaging.

[38] Di Saverio S., Podda M., De Simone B., Ceresoli M., Augustin G., Gori A., Boermeester M., Tolonen M., Bririndelli A., Biffl W. (2020). Diagnosis and treatment of acute appendicitis: 2020 update of the WSES Jerusalem guidelines. World J. Emerg. Surg..

[39] Mederos M.A., Reber H.A., Girgis M.D. (2021). Acute Pancreatitis: A Review. J. Clin. Med..

[40] Laurell H., Hansson L.E., Gunnarsson U. (2020). Diagnostic Strategies and Imaging Utilization in Acute Abdominal Pain. J. Clin. Med..

[41] Ganeshan D., Duong P.T., Probyn L., Lenchik L., McArthur T.A., Retrouvey M., Ghobadi E.H., Desouches S.L., Pastel D., Francis I.R. (2018). Structured reporting in radiology: Impact on clarity, consistency, and clinical decision making. Acad. Radiol..

[42] Klar E., Rahmanian P.B., Bücker A., Hauenstein K., Luther B. (2020). Acute Mesenteric Ischemia: A Vascular Emergency. J. Clin. Med..

[43] Tilsed J.V.T., Casamassima A., Kurihara H., Mariani D., Martinez I., Pereira J., Ponchietti L., Shamiyeh A., Al-Ayoubi F., Barco L.A.B. (2020). ESTES Guidelines: Acute Mesenteric Ischaemia. J. Clin. Med..

[44] Thoeni R.F. (2018). The Revised Atlanta Classification of Acute Pancreatitis: Its Importance for the Radiologist. Diagnostics.

[45] Kahn C.E., Heilbrun M.E., Applegate K.E. (2019). From guidelines to practice: How reporting standards shape radiology communication. Radiology.

[46] American College of Radiology (2020). ACR practice parameter for communication of diagnostic imaging findings. Radiology.

[47] Hsieh H.-F., Shannon S.E. (2022). Three Approaches to Qualitative Content Analysis in Health Research. Diagnostics.

[48] Rubin G.D., Ryerson C.J., Haramati L.B., Sverzellati N., Kanne J.P., Raoof S., Schluger N.W., Volpi A., Yim J.J., Martin I.B.K. (2020). The Role of Chest Imaging in Patient Management during the COVID-19 Pandemic: A Multinational Consensus Statement from the Fleischner Society. Radiology.

[49] Sistrom C.L., Dreyer K.J. (2005). Reporting errors and communication failures in radiology. Radiographics.

[50] Brady A.P. (2017). Error and discrepancy in radiology: Inevitable or avoidable?. Insights Imaging.

[51] Erickson B.J., Korfiatis P., Akkus Z., Kline T.L. (2017). Machine learning for medical imaging. Radiographics.

[52] Topol E.J. (2019). High-performance medicine: The convergence of human and artificial intelligence. Nat. Med..

[53] Mayring P. (2020). Qualitative Content Analysis: Theoretical Foundation, Basic Procedures and Software Solution. J. Clin. Med..

[54] Lombard M., Snyder-Duch J., Bracken C.C. (2020). Content Analysis in Mass Communication: Assessment and Reporting of Intercoder Reliability. J. Clin. Med..

[55] Elo S., Kyngäs H. (2008). The qualitative content analysis process. J. Adv. Nurs..

[56] Schreier M. (2012). Qualitative Content Analysis in Practice.

[57] Guest G., Bunce A., Johnson L. (2006). How many interviews are enough? An experiment with data saturation and variability. Field Methods.

[58] Sandelowski M. (2000). Whatever happened to qualitative description?. Res. Nurs. Health.

[59] Goldberg-Stein S., Chernyak V. (2019). Adding Value in Radiology Reporting. J. Am. Coll. Radiol..

[60] Moher D., Liberati A., Tetzlaff J., Altman D.G. (2009). Preferred reporting items for systematic reviews and meta-analyses: The PRISMA statement. PLoS Med..

[61] Gusenbauer M., Haddaway N.R. (2020). Which academic search systems are suitable for systematic reviews or meta-analyses?. Res. Synth. Methods.

